# Symptomatic Liver Cyst Successfully Treated with Transgastric Drainage and Sclerotherapy Using Minocycline Hydrochloride

**DOI:** 10.1155/2024/6942345

**Published:** 2024-01-17

**Authors:** Kengo Yoshitomi, Yudai Koya, Koichiro Miyagawa, Yuki Maruno, Koki Yamaguchi, Ryuta Taniguchi, Koji Onitsuka, Yoshitaka Sakamoto, Shinji Oe, Masaru Harada

**Affiliations:** ^1^Department of Gastroenterology, Kyushu Rosai Hospital, Moji Medical Center, Kitakyushu, Japan; ^2^The Third Department of Internal Medicine, School of Medicine, University of Occupational and Environmental Health, Kitakyushu, Japan; ^3^Department of Surgery, Kyushu Rosai Hospital, Moji Medical Center, Kitakyushu, Japan

## Abstract

A liver cyst is hepatic fluid-filled cavities often detected in clinical surveillances such as a health examination. Although the liver cyst is usually asymptomatic and observed without any therapeutic intervention, it can be symptomatic and needs treatment due to its enlargement, hemorrhage, and infection. A 74-year-old woman presented with upper abdominal pain and a huge liver cyst in the left lobe. Several examinations including image findings revealed that the symptom could be derived from the liver cyst. Although there is no definite guideline of treatment for symptomatic liver cysts, percutaneous ultrasound-guided drainage with sclerotherapy or surgery is often selected. Because of anatomical accessibility to the liver cyst and the patient's wish, we performed endoscopic transgastric drainage with insertion of both an internal stent and an external nasocystic tube. Sclerotherapy with minocycline hydrochloride was performed through the nasocystic tube, and the liver cyst shrunk completely without any complications. This is the first reported method of administering minocycline hydrochloride through a nasocystic tube, which can be a therapeutic option for patients with symptomatic liver cysts.

## 1. Introduction

Liver cysts are incidentally detected by imaging tests and do not require treatment because most patients are asymptomatic [1]. However, when they develop complications such as obstructive jaundice [2], rupture [3], intracystic hemorrhage [4], or infection [5], invasive treatment is required. Among these complications, liver cyst infection can cause sepsis, which can be fatal [6]. Although there is no evidence-based strategy for the management of liver cyst infection [7], conservative treatment with antibiotics, percutaneous ultrasound-guided drainage, or surgery is usually performed. Antibiotic treatment alone is unlikely to control the infection, and additional treatment such as percutaneous drainage or surgery has been required in 64% of the reported cases [7]. Treatment with aspiration sclerotherapy using ethanol has been reported to have a recurrence rate of up to 21%. On the other hand, most patients show improvement or resolution in symptoms because even partially shrinkage of the liver cyst might be enough to relieve the symptoms [8]. With regard to surgery, recurrence of liver cysts and symptoms occurred in 24% and 22% of the cases, respectively, and reoperation was considered in most cases due to persistent bleeding, thrombosis, or biliary leakage [8]. The percutaneous transhepatic route is commonly selected for infectious liver cyst drainage, followed by sclerotherapy, which is effective in eliminating symptoms in up to 80% of patients [9]. The disadvantages of the percutaneous route include prolonged hospitalization and tube displacement due to body or respiratory movement. Another drainage route is the transluminal route. The effectiveness of this route remains to be fully elucidated, but for enlarged liver cysts located in the left lobe, the transluminal route may be a therapeutic option [10]. Here, we report a case of a symptomatic liver cyst complicated by infection in a patient who responded to sclerotherapy using an external nasocystic tube with minocycline hydrochloride following transgastric drainage.

## 2. Case Report

**Figure 1:**
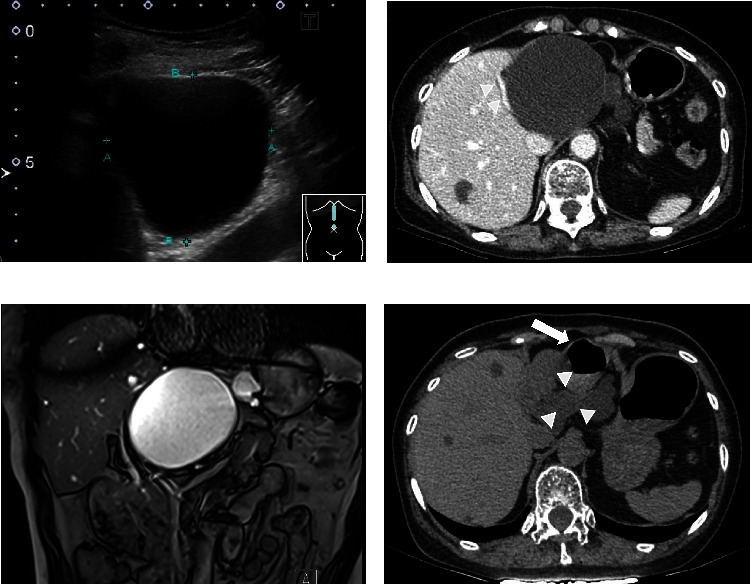
Ultrasonography revealed a giant cyst in the left lobe of the liver (a). The dimensions were 76 × 81 mm. No septa or mural nodules were observed. Contrast-enhanced computed tomography (CT) revealed that the liver cyst had replaced the left lobe. The left branch of the portal vein surrounded the hepatic side of the liver cyst (b) (arrowheads). Magnetic resonance cholangiopancreatography showed that the liver cyst had no communication with the bile duct. There were no neoplastic findings indicating a simple liver cyst (c). On the CT scan after the test puncture (d), the gastric antrum (arrow) migrated to the ventral side of the liver cyst (arrowheads).

**Figure 2:**
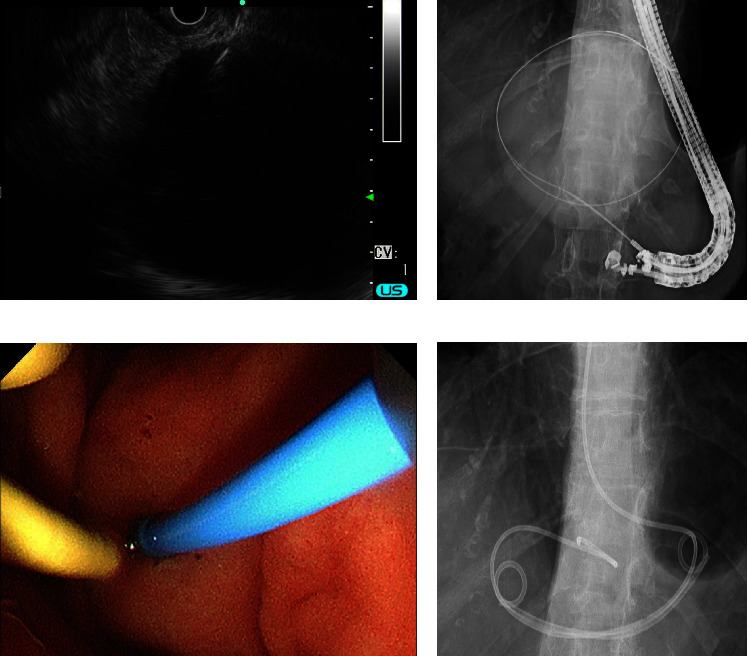
The liver cyst was visualized from the anterior wall of the gastric body using a therapeutic linear echoendoscope and punctured with a 19-gauge needle (a). Two guidewires were inserted into the liver cyst through a cannula (b). An internal 7-Fr loop diameter 10 cm double pigtail catheter and an external 6-Fr nasocystic drainage catheter were inserted into the liver cyst ((c) endoscopy; (d) X-ray).

**Figure 3:**
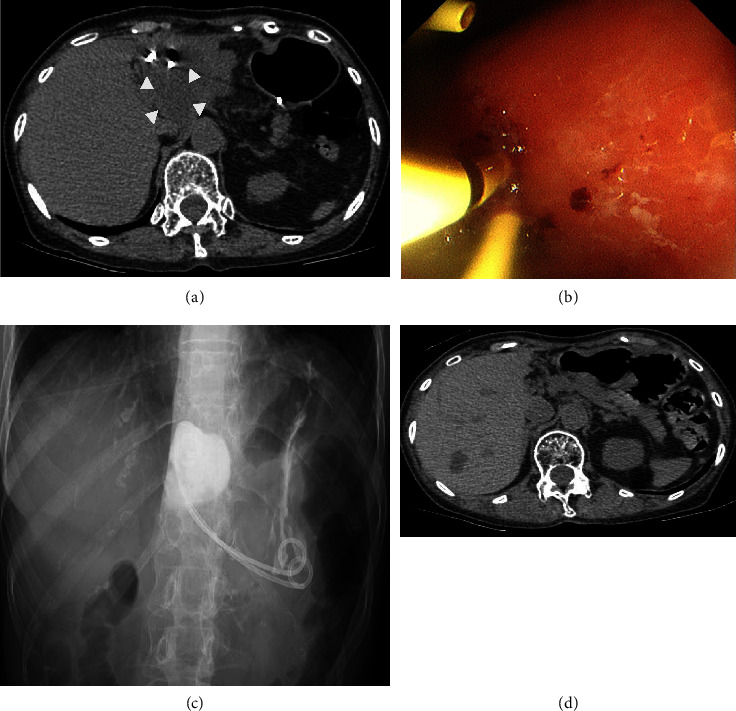
Computed tomography (CT) scan after the first sclerotherapy showing the shrunken liver cyst (a) (arrowheads). We exchanged the external tube for an internal stent (two internal stent insertions) ((b) endoscopy; (c) X-ray). A CT scan showed no recurrence of the liver cyst for 6 months after stent removal (d).

A 74-year-old woman complaining of upper abdominal pain and nausea was admitted to our department. She had a history of hypertension and had been undergoing medical treatment. She was unable to drink alcohol. Physical examination revealed upper abdominal tenderness and a giant mass palpated in the epigastrium. The patient's vital signs were normal. Laboratory test results were within the normal range (Table 1). Abdominal ultrasound (US) revealed several liver cysts, the largest of which was 76 × 81 mm in the left lobe, and a homogeneous anechoic area without septum or mural nodules (Figure 1(a)). Contrast-enhanced computed tomography (CT) showed that the liver cyst occupied the left lobe and broadly compressed the anterior wall of the stomach. In addition, the left branch of the portal vein was shifted to the right side of the liver cyst (Figure 1(b)). Contrast-enhanced CT and magnetic resonance cholangiopancreatography did not show any communication between the bile ducts and the cyst or any findings suggestive of the presence of a tumor (Figures 1(b) and 1(c)). We assumed that the patient's complaint was caused by a giant liver cyst. US-guided percutaneous non-transhepatic aspiration was performed using a 21-gauge needle. Puncture through the liver was not possible as the portal vein was on the puncture line and could not be avoided (Figure 1(b)). The aspirated fluid was yellow transparent with almost normal content data (Table 2), and the tip of the needle became invisible during the procedure. The patient's symptoms completely resolved after treatment. Malignant or tumorous cells were not detected in the aspirated fluid. A CT scan after the puncture revealed that the cyst shrank and the gastric antrum migrated to the ventral side of the liver cyst, which was considered to be the reason for the puncture needle tip's invisibility (Figure 1(d)). The liver cyst subsequently enlarged and returned to its original size within 2 months with exacerbation of symptoms. Radical treatment of the liver cyst was required, and the patient requested nonoperative treatment. There was no room for a transhepatic route on US. The location of the gastric wall and the liver cyst were almost still on CT, so we decided to perform transgastric cyst drainage followed by sclerotherapy. The liver cyst was visualized from the anterior wall of the gastric body using a therapeutic linear echoendoscope (GF-UE 260, Olympus, Tokyo, Japan) and punctured with a 19-gauge needle (EZ Shot 3 Plus, Olympus) (Figure 2(a)). The punctured fluid was slightly turbid and bloody suggesting liver cyst infection. A 0.025-inch guidewire (VisiGlide 2, Olympus) was subsequently inserted into the liver cyst. The fistula was dilated using a 7-Fr dilator (ES Dilator, ZEON MEDICAL, Tokyo, Japan) and a 4 mm dilator balloon (REN, KANEKA MEDIX, Osaka, Japan). Two guidewires were inserted through the cannula (Uneven Double Lumen Cannula, Piolax Medical Devices, Kanagawa, Japan) (Figure 2(b)), an internal 7-Fr loop diameter 10 cm double pigtail catheter (Through Pass, Gadelius Medical, Tokyo, Japan), and an external 6-Fr nasocystic drainage catheter (Flexima ENBD Catheter, Boston Scientific, Massachusetts, USA) (Figures 2(c) and 2(d)). *Enterococcus faecium* was detected in the aspirated fluid culture. Although the patient's symptoms improved after treatment without fever, an inflammatory reaction was elevated (white blood cells: 10,500 counts/*µ*L; C-reactive protein: 40.1 mg/dL). A CT scan showed poor improvement in cyst size, so we considered stent obstruction. Therefore, in addition to intravenous antibiotics, stent replacement was performed, and the inflammatory reaction improved. Although the internal stent was reoccluded within a few days, nasocystic tube patency was preserved with saline lavage. Eleven days after the initial treatment, minocycline hydrochloride (300 mg/day, 60 mL in total with saline) was administered for 4 days through the nasocystic tube. As a result, the liver cyst gradually shrank (Figure 3(a)). Twenty days after the initial treatment, minocycline hydrochloride (100 mg/day, 20 mL in total with saline) was finally administered for 2 days through the nasocystic tube. There were no apparent treatment-related complications during sclerotherapy. We exchanged the nasocystic tube for an internal stent, and the patient was discharged 31 days after the initial treatment with two internal stent placements (Figures 3(b) and 3(c)). The stents were removed 5 months after the initial treatment, and a CT scan showed no recurrence of the liver cyst for 6 months after stent removal (Figure 3(d)).

## 3. Discussion

To the best of our knowledge, this is the first report of a symptomatic liver cyst treated with sclerotherapy using minocycline hydrochloride after endoscopic transgastric drainage. Although the American College of Gastroenterology Clinical Guidelines suggest that symptomatic simple liver cysts should be managed with laparoscopic deroofing rather than aspiration and sclerotherapy [11], sclerotherapy may be useful when accounting for the age and surgical tolerance of patients. Many cases of liver cysts treated with sclerotherapy have been reported [12, 13]; however, there is no consensus regarding the procedural technique and sclerosing agents. Simonetti et al. reported on 35 patients with liver cysts treated with aspiration and sclerotherapy. According to their report, percutaneous aspiration therapy alone had a high recurrence rate, but recurrence was reduced when combined with ethanol sclerotherapy [14]. Percutaneous aspiration with sclerotherapy has minimal adverse events [15], making it a valuable alternative to surgical intervention. A recent systematic review comparing percutaneous aspiration followed by sclerotherapy with surgical treatment for symptomatic simple liver cysts showed that the former was more effective in terms of improving symptoms, recurrence, and complications [15]. In the present case, we initially planned to conduct a test puncture via a percutaneous transhepatic route, but the left branch of the portal vein surrounding the liver cyst disturbed the puncture route (Figure 1(b)). Therefore, the puncture was performed via the percutaneous non-transhepatic route. The cyst was moved away from the abdominal wall by the puncture, and the stomach entered the resulting space, which made it difficult to detect the cyst by ultrasound (Figure 1(d)). The distance between the stomach and the liver cyst did not change during treatment; therefore, we performed transgastric drainage of the liver cyst. There are several reports describing endoscopic ultrasound- (EUS-) guided transluminal drainage for symptomatic liver cysts [10, 16]. A retrospective cohort study of ethanol lavage of huge liver cysts using EUS guidance or a percutaneous approach concluded that EUS-guided transluminal sclerotherapy was also effective and could be a primary method for cysts in the left lobe of the liver [16]. Several advantages for EUS-guided transluminal drainage for a liver lesion have previously been reported, compared to a percutaneous approach: more direct access to the liver lesion in the left lobe through transgastric route, safer puncture by clear visualization of interposed vessels with color Doppler ultrasound, and avoidance of transcutaneous infection [17, 18]. On the other hand, Noh et al. described several limitations: impossibility to visualize and access the right lobe of the liver and requirement of higher technical expertise for EUS-guided transluminal drainage [19]. In addition, stent migration after transluminal drainage of a liver cyst is also necessary to care about, although there have been no reports describing about that. An accumulation of the cases is required to elucidate the efficacy and safety of the EUS-guided transluminal drainage for a liver lesion. There are several sclerosing agents available, ethanol, polidocanol, minocycline hydrochloride [20], and ethanolamine oleate [21]. As our patient was intolerant to ethanol, minocycline hydrochloride was used for sclerotherapy. Since alcohol intolerance has been observed at high percentages in Asian populations, especially in Japan [22, 23], minocycline hydrochloride is useful. Minocycline hydrochloride has been widely used for sclerotherapy of symptomatic liver cysts because of its safety, low cost, and low recurrence rate [20, 24]. One of the mechanisms of action is considered to be its strong acidity (pH 2.0–3.5) which can damage the cystic epithelium [25]. Initially, there was concern about gastric mucosal damage due to reflux of minocycline into the stomach, but since the pH of gastric acid ranges around 1 after eating [26], leakage of minocycline hydrochloride into the stomach was considered safe. As a precaution, oral proton pump inhibitors and sodium alginate were administered to the patient. In addition, minocycline hydrochloride was administered via the nasocystic tube after fistula formation to prevent leakage of the sclerosing agent into the abdominal cavity. We carefully continued the treatment after confirming that there was no gastric mucosal injury or leakage of the agent. In line with the patient's laboratory data, imaging findings, and some reports [27, 28], we placed both internal and external drainage tubes, expecting effective drainage. Considering the mechanism of minocycline hydrochloride causing cyst wall damage, gastric acid itself may also damage and shrink liver cysts with a long duration of internal stent placement. A neutrophil-to-lymphocyte ratio (NLR) is useful to predict the worsening inflammation in several diseases. The NLR was significantly higher in patients with severe infection than in controls, and persistent high NLR led to a poor prognosis in patients with sepsis [29]. Another study reported that high NLR in patients with liver cirrhosis might be associated with the onset of spontaneous bacterial peritonitis [30]. Although the cutoff value of the NLR has not been established yet, an intensive therapy could be taken into consideration in patients with an exacerbation of the NLR [29]. In the present case, the NLR was 2.54 before drainage, but was elevated gradually after drainage, and we found stent obstruction with infection. After exchanging the stents, the NLR was back to the initial level. The NLR might be a biomarker of worsening inflammation and be an indicator of therapeutic efficacy. For this hypothesis, the accumulation of cases and further investigation are required. As for the limitation of this report, there is a risk for internal stent obstruction or cyst infection due to the communication with the stomach like the present case. A stent migration into the liver cyst might occur. To avoid these complications, frequent saline lavage of an external tube and an internal stent which is as long as possible should be inserted. We successfully treated a symptomatic liver cyst with sclerotherapy using minocycline hydrochloride after endoscopic transgastric drainage. If the cyst is located in the left lobe of the liver and the distance between the stomach and the liver cyst is stable, sclerotherapy using minocycline hydrochloride after endoscopic transgastric drainage may be one of the therapeutic options.

## Figures and Tables

**Table 1:** Laboratory data on admission.

*Hematology*	
WBC	5,700 (*μ*L)
RBC	403 × 10^4^ (*μ*L)
Hb	12.9 g/dL
Ht	39.3%
Plt	18.6 × 10^4^ (*μ*L)

*Biochemistry*	
TP	7.5 g/dL
Alb	4.4 g/dL
T-bil	0.62 mg/dL
AST	27 IU/L
ALT	20 IU/L
LDH	263 IU/L
ALP	181 IU/L
GGT	14 IU/L
BUN	13.2 mg/dL
Cre	0.51 mg/dL
Na	142 mEq/L
K	4.3 mEq/L
FPG	70 mg/dL

*Serology*	
CRP	0.18 mg/dL

*Coagulation*	
PT%	116%
PT-INR	0.93
APTT	28.2 sec

*Viral markers*	
HBsAg	(−)
HBcAb	(−)
HCVAb	(−)

*Tumor markers*	
CEA	6.2 ng/mL
CA19-9	38.2 U/mL

RBC: red blood cell, WBC: white blood cell, Hb: hemoglobin, Ht: hematocrit, Plt: platelet, TP: total protein, Alb: albumin, T-bil: total bilirubin, AST: aspartate aminotransferase, ALT: alanine aminotransferase, LDH: lactate dehydrogenase, ALP: alkaline phosphatase, GGT: *γ*-glutamyltransferase, BUN: blood urea nitrogen, Cre: creatinine, Na: natrium, K: kalium, FPG: fasting plasma glucose, CRP: C-reactive protein, PT%: prothrombin time, APTT: activated partial thromboplastin time, HBsAg: HBs antigen, HBcAb: HBs antibody, HCVAb: HCV antibody, CEA: carcinoembryonic antigen, and CA19-9: carbohydrate antigen 19-9.

**Table 2:** Liver cyst content data.

*Hematology*	
WBC	<100 (*μ*L)

*Biochemistry*	
TP	1.1 g/dL
Alb	0.5 g/dL
T-bil	0.07 mg/dL
LDH	263 IU/L

*Tumor markers*	
CEA	20.8 ng/mL
CA19-9	79.9 × 10^4^ U/mL

WBC: white blood cell, TP: total protein, Alb: albumin, T-bil: total bilirubin, LDH: lactate dehydrogenase, CEA: carcinoembryonic antigen, and CA19-9: carbohydrate antigen 19-9.

## Data Availability

The data used to support this study are included within the article.
